# Effect of oblique headless compression screw fixation for metacarpal shaft fracture: a biomechanical in vitro study

**DOI:** 10.1186/s12891-020-03939-2

**Published:** 2021-02-05

**Authors:** Yung-Cheng Chiu, Tsung-Yu Ho, Yen-Nien Ting, Ming-Tzu Tsai, Heng-Li Huang, Cheng-En Hsu, Jui-Ting Hsu

**Affiliations:** 1 School of Medicine, China Medical University, Taichung, 404 Taiwan; 2 Department of Orthopedic Surgery, China Medical University Hospital, Taichung, 404 Taiwan; 3 3D Printing Medical Research Center, China Medical University Hospital, Taichung, 404 Taiwan; 4 Department of Biomedical Engineering, Hungkuang University, Taichung, Taiwan, Republic of China 433; 5 School of Dentistry, College of Dentistry, China Medical University, 91 Hsueh-Shih Road, Taichung, 40402 Taiwan; 6 Department of Bioinformatics and Medical Engineering, Asia University, Taichung, 413 Taiwan; 7 Department of Orthopaedics, Taichung Veterans General Hospital, No. 1650, Sec. 4 Taiwan Boulevard, Situng Dist., Taichung City, 407 Taiwan (Republic of China); 8 Sports Recreation and Health Management Continuing Studies-Bachelor’s Degree Completion Program, Tunghai University, Taichung, 407 Taiwan

**Keywords:** Metacarpal shaft fracture, Bone plate, Compression screw

## Abstract

**Background:**

Metacarpal shaft fracture is a common fracture in hand trauma injuries. Surgical intervention is indicated when fractures are unstable or involve considerable displacement. Current fixation options include Kirschner wire, bone plates, and intramedullary headless screws. Common complications include joint stiffness, tendon irritation, implant loosening, and cartilage damage.

**Objective:**

We propose a modified fixation approach using headless compression screws to treat transverse or short-oblique metacarpal shaft fracture.

**Materials and methods:**

We used a saw blade to model transverse metacarpal neck fractures in 28 fresh porcine metacarpals, which were then treated with the following four fixation methods: (1) locked plate with five locked bicortical screws (LP group), (2) regular plate with five bicortical screws (RP group), (3) two Kirschner wires (K group), and (4) a headless compression screw (HC group). In the HC group, we proposed a novel fixation model in which the screw trajectory was oblique to the long axis of the metacarpal bone. The entry point of the screw was in the dorsum of the metacarpal neck, and the exit point was in the volar cortex of the supracondylar region; thus, the screw did not damage the articular cartilage. The specimens were tested using a modified three-point bending test on a material testing system. The maximum fracture forces and stiffness values of the four fixation types were determined by observing the force–displacement curves. Finally, the Kruskal–Wallis test was adopted to process the data, and the exact Wilcoxon rank sum test with Bonferroni adjustment was performed to conduct paired comparisons among the groups.

**Results:**

The maximum fracture forces (median ± interquartile range [IQR]) of the LP, RP, HC, and K groups were 173.0 ± 81.0, 156.0 ± 117.9, 60.4 ± 21.0, and 51.8 ± 60.7 N, respectively. In addition, the stiffness values (median ± IQR) of the LP, HC, RP, and K groups were 29.6 ± 3.0, 23.1 ± 5.2, 22.6 ± 2.8, and 14.7 ± 5.6 N/mm, respectively.

**Conclusion:**

Headless compression screw fixation provides fixation strength similar to locked and regular plates for the fixation of metacarpal shaft fractures. The headless screw was inserted obliquely to the long axis of the metacarpal bone. The entry point of the screw was in the dorsum of the metacarpal neck, and the exit point was in the volar cortex of the supracondylar region; therefore the articular cartilage iatrogenic injury can be avoidable. This modified fixation method may prevent tendon irritation and joint cartilage violation caused by plating and intramedullary headless screw fixation.

## Introduction

Metacarpal shaft fractures account for the second highest number of metacarpal fractures, following only metacarpal neck fractures. Metacarpal shaft fractures and metacarpal neck fractures occur at a ratio of 1:2 [1]. Metacarpal shaft fractures commonly occur due to axial loading, torsion, or direct blows. Fractures can be classified as oblique, transverse, or comminuted. Axial loading and direct blows often cause transverse or comminuted fractures, whereas torsion commonly results in oblique or spiral fractures. Oblique and spiral fractures, which account for approximately 75% of fracture cases, are the most common [2]. Most nondisplaced fracture cases are treated using cast immobilization. The indications for surgery are unstable fractures or overlapping fractured bone ends that cause excessive shortening of bone length, angulation deformity, or the rotation deformity of fracture sites [3]. In short-oblique or transverse metacarpal shaft fractures, fracture sites are subject to angulation deformity because interosseous muscles exert traction force on the fracture site. Furthermore, because of the limited bone contact area at the fractured bone end, the fracture site is relatively unstable, and nonunion of fracture is not uncommon. Therefore, surgical intervention is often required [4].

Common surgical methods for metacarpal shaft fracture fixation include (1) Kirschner wire (K-wire) fixation, (2) regular plate fixation, and (3) locked plate fixation. Studies and clinical experience have yet to determine the optimal fixation method [5–8]. K-wire fixation predominantly involves minimally invasive surgical procedures. However, its mechanical stability and potential complications, such as K-wire breakage, fracture site loss reduction, and infection at K-wire entry sites, are of concern [9–15]. To prevent surgical fixation failures, patients with interfragmentary K-wire fixation are often recommended to undergo immobilization for 6–8 weeks before starting a rehabilitation program. However, prolonged immobilization may result in the sequela of finger joint stiffness. By contrast, bone plate fixation achieves greater mechanical stability [16–20], shortens the required postoperative immobilization period, and enables patients to immediately initiate rehabilitation programs [8, 9]. However, bone plate fixation, whether locked or regular, also has disadvantages such as postoperative metacarpophalangeal joint stiffness, extensor tendon adhesion, and higher surgical costs than other methods; moreover, it may cause iatrogenic injury to the dorsal cutaneous branch of the ulnar nerve or necessitate a secondary surgery for plate removal [5, 15, 19, 21–23].

To address the unsatisfactory fixation effects of K-wire fixation, several researchers reported on the use of intramedullary headless screws [24–27]. Avery et al. [25] indicated that compared with K-wire fixation, headless compression screws for metacarpal neck fractures exhibited biomechanically superior performance in terms of load to failure, three-point bending, and axial loading. Furthermore, Siddiqui et al. reported that patients with intramedullary screw fixation for metacarpal fractures had favorable postoperative results with early return to work. However, this minimally invasive surgical method has the risk of potentially damaging the articular cartilage. Studies have indicated that the destruction of the articular cartilage of finger joints has the inevitable sequelae of pain and stiffness as well as arthritis in finger joints [28, 29].

The present study proposes a fixation method that employs headless compression screws and generates fixation strength similar to that of interfragmentary compression. This study aimed to further enhance the stability of the proposed method to a level similar to that of bone plate fixation, avoid the disadvantages of exposed screw heads in bone plate fixation and conventional lag screw fixation, and, most importantly, prevent irreversible sequelae caused by articular cartilage destruction.

## Materials and methods

### Specimen preparation

We used 28 fresh frozen porcine fifth metacarpals in this study because it is difficult to obtain an adequate number of fresh human specimens with identical bone quality and size. The fresh frozen porcine fifth metacarpal specimens were obtained from local meat market. All pigs were male, approximately 2 years of age, and 110 kg in weight. A metacarpal shaft fracture was generated using a 0.4-mm saw blade. The fracture distance for all specimens was 25 mm from the distal articular surface.

### Fixation approaches

The specimens were assigned to four fixation techniques, all of which were performed by a single senior hand surgeon (Yung-Cheng Chiu). Details concerning the techniques applied to the various groups are provided in the following bullets.

**Fig. 1:**
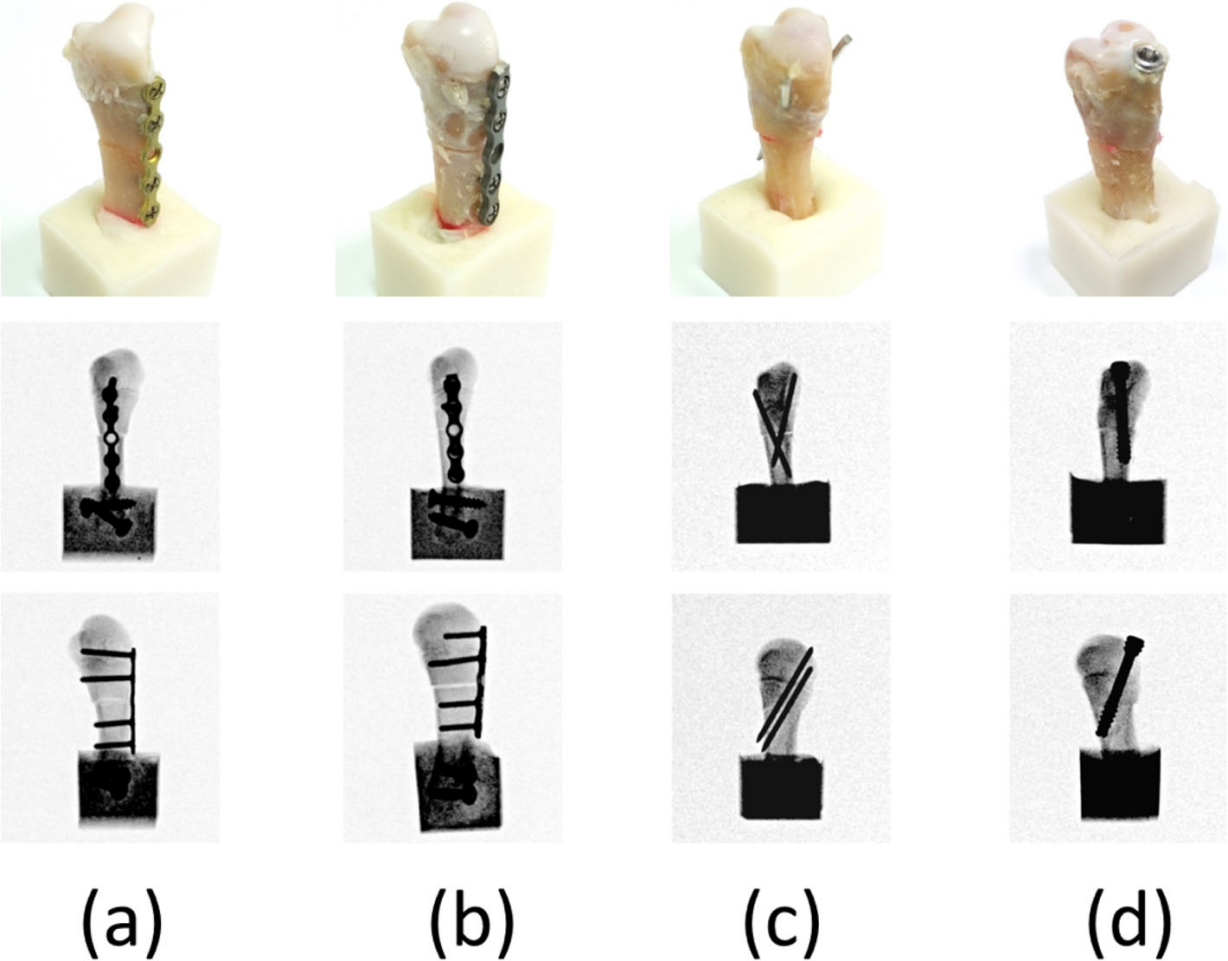
Porcine fifth metacarpals and four fixation types for metacarpal shaft fracture. **a** Straight locked plate with four locked bicortical screws (LP group). **b** Straight regular plate with four bicortical screws (RP group). **c** Cross-pin fixation of two K wires (K group). **d** Headless compression screw (HC group). Radiographs of the four fixation types are also shown in this figure (upper row: anterior–posterior view; bottom row: lateral view)

**Fig. 2:**
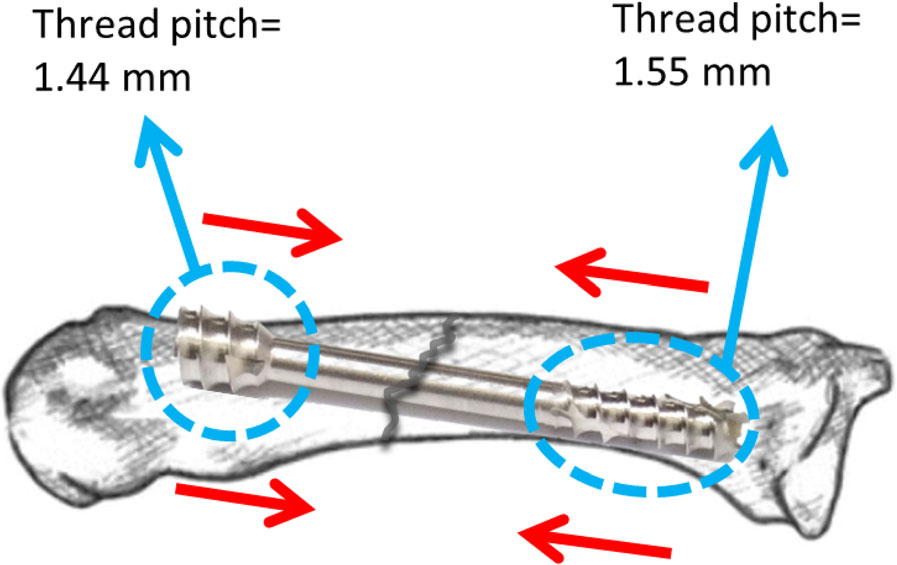
The modified headless compression screw approach was used to fix the metacarpal shaft fracture; the screw trajectory was oblique to the long axis of the metacarpal

- Group 1 (LP group): Locked plate with five holes and four locked bicortical screws. Seven specimens were fixed using the straight five-hole locked plate with four 2.3-mm diameter locked screws (Stryker, Germany). The locked plates were applied at the dorsum of the metacarpal shaft with two bicortical locked screws fixed distally to the fracture site and two bicortical locked screws fixed proximally to the fracture site (Fig. 1a).
- Group 2 (RP group): Regular plate with five holes and four bicortical screws. Seven specimens were fixed using the straight nonlocked plate with four 2.3-mm diameter compression screws (Stryker, Germany). The nonlocked plates were applied at the dorsum of the metacarpal shaft with two bicortical compression screws fixed distally to the fracture site and two bicortical compression screws fixed proximally to the fracture site (Fig. 1b).
- Group 3 (K group): Two K wires. Seven specimens were stabilized with two 1.5-mm-diameter K wires inserted distally from the dorsal medial and on the lateral side of the metacarpal neck; they penetrated through the fracture site and proximally punctured out from the proximal volar cortex during cross-pin fixation. Fracture reduction was maintained with manual axial compression during the surgery (Fig. 1c).
- Group 4 (HC group): Seven specimens were stabilized with a single 4.3-mm Dart-fire headless screw (USA, Wright) with wires inserted from the dorsal metacarpal head that penetrated through the fracture site and punctured out from the proximal volar cortex. The fracture reduction was maintained through manual axial compression during fixation of the headless compression screw (Figs. 1d and 2).

### Biomechanical test

**Fig. 3:**
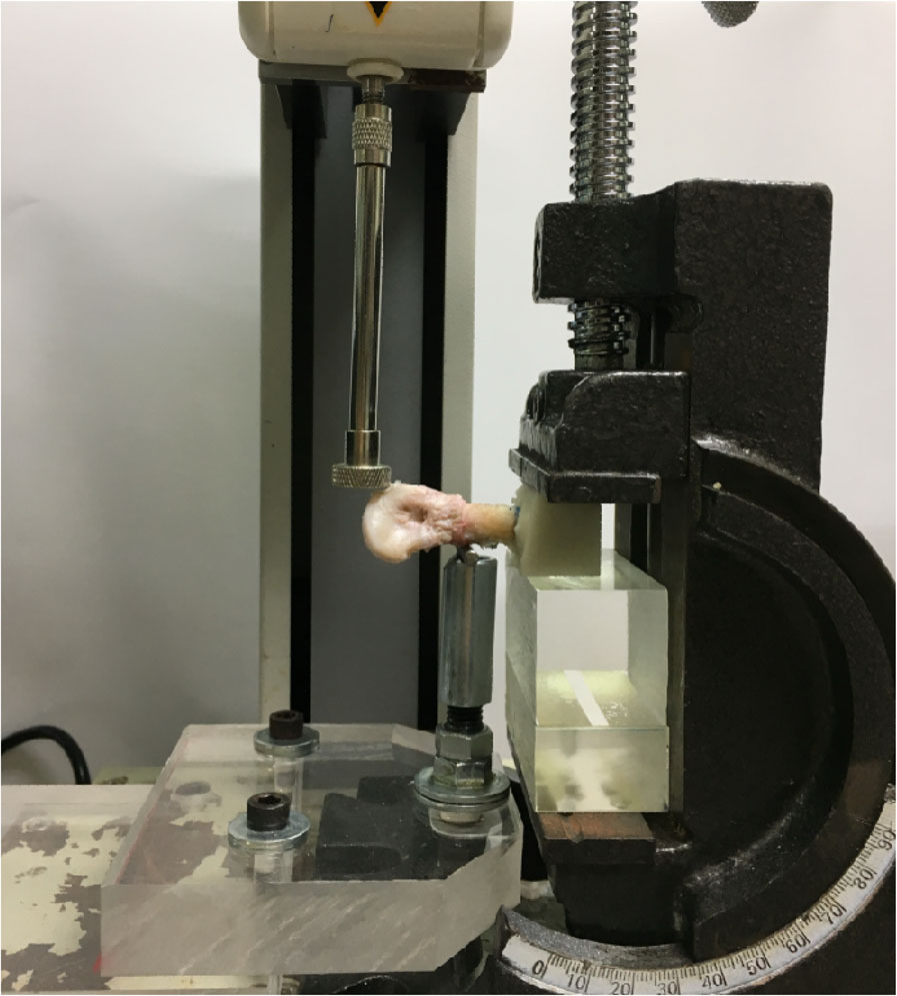
Experimental setup for biomechanical testing: modified three-point bending test

The biomechanical testing setup was a modified three-point bending test [15, 30, 31]. Briefly, the proximal end of each specimen was held in a custom fixture with bone cement clamps before biomechanical testing. A volar support was centered under each specimen (5 mm proximal from the fracture site). The biomechanical tests were conducted using a material testing system (JSV-H1000, Japan Instrumentation System, Nara, Japan; Fig. 3). The perpendicular load of 10 mm/min was applied on the dorsal side of the specimen 53 mm from the fixed point of the fracture. The raw data for force–displacement were recorded, and the maximum fracture and bending stiffness were determined for each specimen.

### Statistical analysis

Statistical analyses were performed using SPSS Version 19 (IBM Corporation, Armonk, NY, USA). The maximum fracture forces and the stiffness values of the four fixation types were summarized as median ± interquartile range (IQR). The Kruskal–Wallis test was used to compare the maximum fracture force and stiffness measurements of the four groups. Post hoc pairwise comparisons were conducted using the exact Wilcoxon rank sum test with Bonferroni adjustment. Results with *P* < 0.00833 (=0.05/6) were considered significant.

## Results

**Fig. 4:**
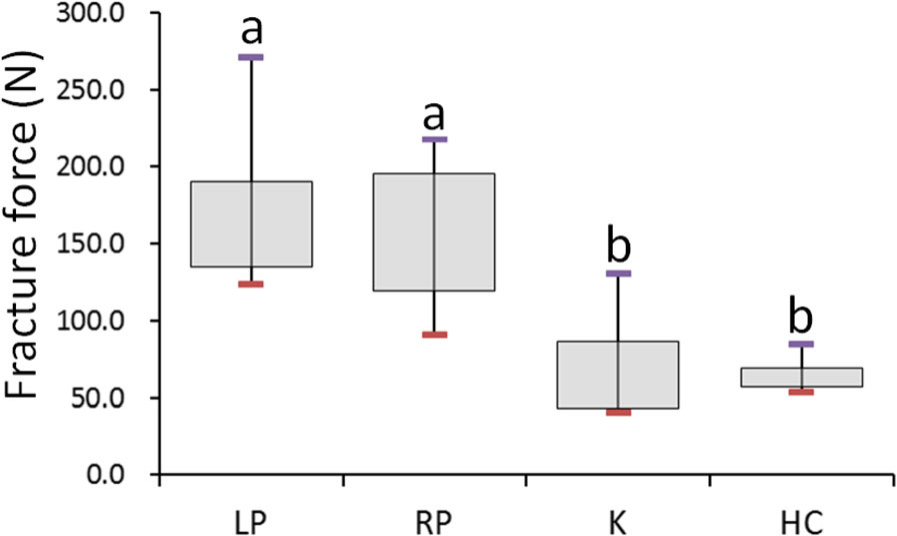
Box plot showing the fracture forces of the four fixation types. Post hoc pairwise comparisons were conducted using the exact Wilcoxon rank sum test with Bonferroni adjustment; same letters indicate that medians were not significantly different at the 0.00833 (0.05/6) level. LP: locked plate with five locked bicortical screws; RP: regular plate with five bicortical screws; K: two K wires; HC: headless compression screw

The maximum fracture forces of the four fixation types are listed in Table 1. The highest maximum fracture force among the four fixation types was in the LP group (173.0 ± 81.0 N. The median maximum fracture force in the RP group (156.0 ± 117.9 N) was slightly lower than that of the LP group (Fig. 4). In addition, the maximum fracture force in the K group (51.8 ± 60.7 N) was slightly higher than that in the HC group (60.4 ± 21.0 N; Fig. 4). The maximum fracture force of the LP and RP groups was significantly higher than that of the K and HC groups.

**Table 1:** Maximum fracture force (N) of the four fixation types for metacarpal shaft fracture

Group	Sample size	Median	IQR	MAX	MIN	*P*^a^
LP	7	173.0	81.0	270.8	123.6	< 0.001
RP	7	156.0	117.9	217.5	90.7	
K	7	51.8	60.7	130.4	40.7	
HC	7	60.4	21.0	84.9	53.3	

*LP* locked plate with five locked bicortical screws, *RP* regular plate with five bicortical screws, *K* two K wires, *HC* headless compression screw

^a^Kruskal–Wallis test

**Fig. 5:**
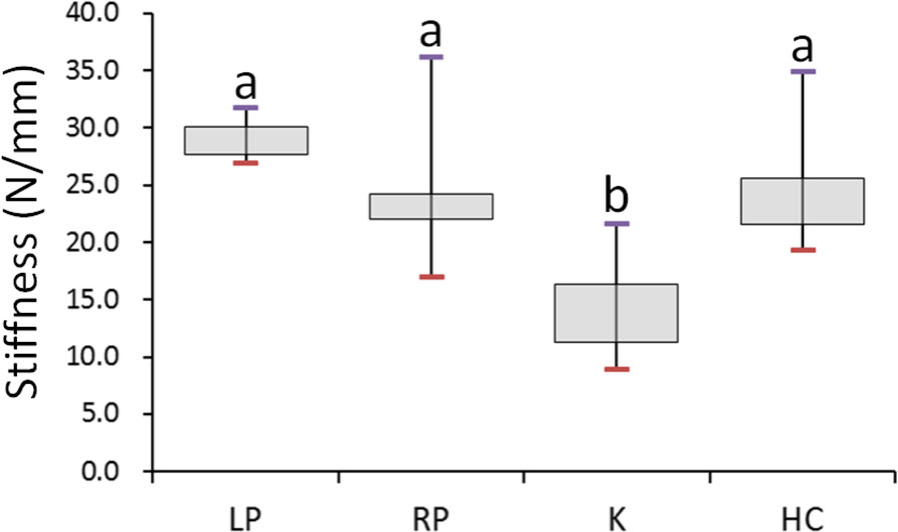
Box plot showing the stiffness values of the four fixation types. Post hoc pairwise comparisons were conducted using the exact Wilcoxon rank sum test with Bonferroni adjustment; same letters indicate that medians were not significantly different at the 0.00833 (0.05/6) level. LP: locked plate with five locked bicortical screws; RP: regular plate with five bicortical screws; K: two K wires; HC: headless compression screw

The stiffness values of the four fixation types are listed in Table 2. The highest stiffness among the four fixation types was in the LP group and was 29.6 ± 3.0 N/mm (Fig. 5). The median stiffness of the RP group (22.6 ± 2.8 N/mm) was slightly lower than that of the HC group (23.1 ± 5.2 N/mm; Fig. 5). However, no significant differences were observed between the LP, RP, and HC groups. The stiffness of the K group (14.7 ± 5.6 N/mm) was the lowest (Fig. 5).

**Table 2:** Stiffness (N/mm) of the four fixation types for metacarpal shaft fracture

Group	Sample size	Median	IQR	MAX	MIN	*P*^a^
LP	7	29.6	3.0	31.7	26.9	< 0.001
RP	7	22.6	2.8	36.2	16.9	
K	7	14.7	5.6	21.6	8.9	
HC	7	23.1	5.2	34.9	19.2	

*LP* locked plate with five locked bicortical screws, *RP* regular plate with five bicortical screws, *K* two K wires, *HC* headless compression screw

^a^Kruskal–Wallis test

## Discussion

Metacarpal shaft fracture is a common hand fracture. Current internal fixation methods include use of K-wire, bone plate, and intramedullary headless screw fixation. However, these surgical treatments have disadvantages such as pin tract infections, poor wound healing or wound dehiscence, stiffness of the finger joint, adhesion of the extensor tendon, high cost of bone plates, and a necessary secondary surgery for plate removal. To avoid these disadvantages, we proposed the modified headless compression screw method. First, we insert the screw in an oblique trajectory. Because the screw does not enter through the articular cartilage, the potential complication of articular cartilage destruction caused by intramedullary screw fixation is avoided. Furthermore, because the screw head is almost embedded in bone and has limited contact with the extensor tendon, extensor tendon adhesion caused by bone plate fixation is prevented. After patients recover from fractures, they do not require secondary surgery for plate removal. In addition, the proposed method is considerably cheaper than bone plate fixation surgery. Despite the aforementioned advantages, the modified method’s stability in fracture fixation is uncertain. Therefore, we employed porcine bones to verify its stability for transverse metacarpal shaft fractures. The test results revealed that the oblique headless compression screw method exhibited stiffness similar to that exhibited by the locked plate and regular plate fixation methods and significantly higher stiffness than that of the K-wire fixation method.

Because fresh cadaver metacarpals are difficult to obtain, some scholars have used artificial bones for testing [32–34]. Although the use of artificial bones means that each bone specimen has the identical shape and material properties, artificial bones cannot replicate the trabecular bone structure and anisotropic properties found in actual bones. Therefore, porcine metacarpals, which are highly similar to human metacarpals, were employed for testing. Past studies have also employed porcine metacarpals for biomechanical tests [15, 21, 30, 31]. According to the experimental results, the coefficients of variation for the LP, RP, and K groups were all approximately 30%. These coefficients were higher than those of our previous study, in which artificial bones were employed to investigate the stability of the three fixation methods in metacarpal neck fractures [34]. This may have been because of differences between the porcine metacarpal specimens.

The modified three-point bending test was adopted for this study, but this test is not completely identical to the physiological loading in common clinical scenarios. As a practical matter, no in vitro biomechanical tests can authentically reflect physiological loadings. In addition to the modified three-point bending test [15, 30, 31], cantilever bending tests [32, 35], three-point bending tests [36], four-point bending tests [19, 37], and torsional tests [32, 38] have been adopted in previous studies. In this study, the cantilever bending test was not used because our pilot experiment revealed that the proximal end of the metacarpal bone was removed from the custom fixture with bone cement clamps during the cantilever bending loading. Therefore, per the methods of other studies [15, 30, 31], the modified three-point bending test was used in this study. In addition, as in other studies, we employed the maximum fracture force and stiffness as indicators of stability [19, 21, 30, 32, 36, 38]. However, we prioritized the stiffness indicator over the maximum fracture force indicator because during the bone healing process, patients should not and are unlikely to experience a refracture in the metacarpal shaft caused by extreme active or passive forces. Furthermore, the stiffness indicator represents the structural stiffness of the fixed fracture site. Therefore, stiffness evaluation should be prioritized over maximum fracture force.

K-wire fixation surgery is the approach with the lowest cost. Compared with bone plates, K wires fixation approach also requires less soft tissue dissection [5], thereby reducing the occurrence of postoperative extensor tendon adhesion. However, K-wire fixation has potential disadvantages such as reduced resistance to fracture causing fixation failure, pin tract infection, and breakage of pin due to metal fatigue. Other disadvantages include a prolonged immobilization period due to weak fixation strength [9, 10]. In recent years, the use of locked bone plates has substantially improved fracture treatment. Because locked plates provide stronger stability, patients are able to move with their injured extremity and launch the rehabilitation program at an earlier stage, thus reducing the duration of sick leave and allowing patients to recover more satisfactory joint range of motion [8]. Because the dorsal side of the metacarpal bone is covered by thin skin and because the extensor digitorum tendon is attached to the bone, bone plate fixation may cause metacarpophalangeal joint stiffness and extensor tendon adhesion. Additionally, the long surgical incision easily leads to iatrogenic injury in the dorsal branch of the ulnar nerve. Because of discomfort at the fracture site, patients must undergo a secondary surgery for implant removal after bone healing [22, 23]. Locked bone plate fixation provides more favorable fracture fixation effects. However, due to complications following bone plate fixation, consensus has yet to be reached regarding whether bone plate fixation is more satisfactory than K-wire fixation for treating metacarpal shaft fractures [23].

In other studies, researchers have indicated that the intramedullary headless compression screw outperforms the K-wire in fixation. Furthermore, compared with bone plate fixation on the dorsal side, the intramedullary headless compression screw prevents metacarpophalangeal joint stiffness and extensor tendon adhesion. However, the intramedullary headless compression screw can damage the articular cartilage, causing arthritic changes. Therefore, in this study, we proposed a modified fixation approach for metacarpal shaft fractures in which a headless compression screw is inserted obliquely. The screw enters the dorsal cortex of the metacarpal neck and exits the volar cortex of the supracondylar region. Because the screw entry point is distanced from the articular cartilage, the risk of cartilage destruction caused by intramedullary screw fixation can be avoided. The results of this study are consistent with those of previous studies [36, 39, 40]. The maximum fracture forces and stiffness values of the LP and RP groups were higher than those of the K group. The maximum fracture force of the HC group did not differ significantly from that of the K group and was lower than that of the LP and RP groups. However, during clinical bone healing, the structure stiffness of the fixation for the metacarpal shaft fracture was more influential than the maximum fracture force. The experimental results indicated that the stiffness of the HC group was nonsignificantly different from that of the LP and RP groups. This indicates that the HC group has a similar stiffness to that of the RP and LP groups. Clinically, stiffness is more crucial than maximum fracture force.

In this study, we proposed a fixation method that uses a headless screw to generate interfragmentary compression (HC group). Compared with bone plate fixation, the proposed method is less invasive; has similar mechanical strength; and avoids complications such as postoperative metacarpophalangeal joint stiffness, adhesion of the extensor tendon, iatrogenic injury of the dorsal cutaneous branch of the ulnar nerve, and the necessity of a secondary surgery for plate removal [5, 15, 19, 21–23]. The proposed method not only has surgical costs considerably lower than those of bone plate fixation and other methods but also avoids the disadvantage of prominent screw heads, which may cause tendon adhesion and discomfort at the surgical site. Furthermore, because the headless compression screw is also the design of cannulated screw, a pilot K wire must first be used to confirm the screw trajectory before final tightening. Therefore, the proposed fixation method is more precise and safer than conventional compression lag screw fixation.

This study had the following limitations. First, because fresh human metacarpal bones are difficult to obtain, the experiment design adopted porcine metacarpals with reference to the literature [15, 21, 30, 31]. Fresh animal bones have trabecular bone structure and anisotropic and inhomogeneous properties, and the strength and shape of porcine metacarpals differ from those of human metacarpals. Second, we employed the modified three-point bending test for stability measurement [15, 30, 31]. This loading mode cannot simulate actual physiological conditions. Furthermore, artificial bones and animal bone specimens do not contain soft tissues such as muscles, ligaments, and tendons. This limitation also affected previous studies [37, 41]. Nevertheless, porcine metacarpals were adopted to compare the stabilities of the four fixation methods. Although discrepancies exist between maximum fracture force and stiffness values tested in this study and those in clinical applications on the human body, these limitations did not influence the comparison of stability among the four fixation methods.

## Conclusion

In this study, we proposed a modified fixation method for metacarpal shaft fractures, whereby a headless compression screw is inserted obliquely. The screw enters from the dorsal cortex of the metacarpal neck and exits from the volar cortex of the supracondylar region. Because the screw entry point is not on the articular cartilage, the potential complication of cartilage destruction that caused by intramedullary screw fixation can be avoided. The in Vitro biomechanical experiments indicated that the maximum fracture force of the proposed fixation method was less than that in bone plate fixation. However, the stiffness, which is a superior evaluation index for bone fracture healing time, for the proposed method was similar to that for bone plate fixation.

## Data Availability

All data generated or analysed during this study are included in this published article [and its supplementary information files].
